# Restoring bone healing potential

**DOI:** 10.7554/eLife.105420

**Published:** 2025-01-13

**Authors:** Inês Sequeira

**Affiliations:** 1 Centre for Oral Immunobiology and Regenerative Medicine, Institute of Dentistry, Queen Mary University of London London United Kingdom; 2 Barts Centre for Squamous Cancer, Queen Mary University of London London United Kingdom

**Keywords:** osteoprogenitors, aging, intermittent fasting, bone, Wnt, bone regeneration, Mouse

## Abstract

A combination of intermittent fasting and administering Wnt3a proteins to a bone injury can rejuvenate bone repair in aged mice.

**Related research article** Reeves J, Tournier P, Becquart P, Carton R, Tang Y, Vigilante A, Fang D, Habib SJ. 2024. Rejuvenating aged osteoprogenitors for bone repair. *eLife* **13**:RP104068. doi: 10.7554/eLife.104068.

As we become older our body experiences a progressive decline in its ability to heal and regenerate. Bones are particularly affected (Colón et al., 2018), with age-related changes decreasing the number and functionality of osteoprogenitors – the cells that are required for bone development and repair. Aging can also decrease the blood supply to the bones and reduce mitochondrial activity, which is necessary for generating energy in cells (Clark et al., 2017).

Flat bones – such as the sternum, scapula, pelvis and skull – play crucial roles in protecting organs and anchoring muscles. However, their thin structure makes them prone to fractures (Huelke and Compton, 1983), and this risk increases as they become more brittle with age (Beedham et al., 2019). Skull fractures resulting from head injuries are particularly concerning in older individuals (Tsai et al., 2022).

As the global population ages, the need for innovative strategies to promote bone healing in elderly people becomes more urgent; however, previous approaches – including those based on growth factors, biomaterials, and cell therapies – have fallen short. This may be due to the bone tissue microenvironment – the surrounding cells, molecules and structures that support bone formation and repair – deteriorating, becoming stiffer, and displaying reduced metabolic activity with age.

Emerging evidence suggests that targeting metabolic pathways and rejuvenating the bone microenvironment could unlock the regenerative potential of aged tissues. However, this has been limited by a lack of studies on mice that are old enough to accurately reflect the decline seen in elderly humans. Now, in *eLife*, Shukry Habib (University of Lausanne) and colleagues – including Joshua Reeves as first author – report a detailed investigation of bone regeneration in very old mice (Reeves et al., 2024).

The researchers – who are at Lausanne, King’s College London and Zhejiang University – studied mice that were over 20 months old, which is equivalent to approximately 70 human years (Dutta and Sengupta, 2016). To identify systemic and local interventions that can restore the microenvironment of aged bones and also support bone healing, Reeves et al. employed a dual therapeutic approach to treat a bone injury in the skull of aged mice.

A bandage was used to administer a signalling protein called Wnt3a (which activates osteoprogenitors and thus drives bone growth and formation) to the injury site. This was combined with an intermittent fasting regimen, a systemic intervention known to enhance mitochondrial function and vascular health (Catterson et al., 2018; Longo and Anderson, 2022). Remarkably, this combination increased the number of osteoprogenitors at the site of injury and also restored their functionality, resulting in bone healing outcomes comparable to those seen in young mice.

At the cellular level, Reeves et al. provide fascinating insights into why this combined approach has a better outcome than either strategy alone. Amongst other benefits, intermittent fasting was shown to improve mitochondrial activity – while also improving the vascularisation of the tissue. This is critical for providing bone-healing cells with oxygen and nutrients – something which can be compromised as blood vessels deteriorate with age. Intermittent fasting also enhanced the production of NAD^+^ (a molecule which is vital for cellular energy and repair) in osteoprogenitor cells and improved the balance of beneficial bacteria in the gut of aged mice.

The Wnt3a bandage, on the other hand, boosted the number of osteoprogenitor cells at the site of injury. However, these cells only regained full functionality with intermittent fasting. Similar bone-healing benefits were also achieved without fasting by either directly increasing NAD^+^ levels through supplements, or by specifically modifying the gut bacteria population.

Taken together, these findings highlight the critical role of metabolic health in bone repair, as well as demonstrating the importance of integrating both systemic and local strategies for effective bone regeneration. Previous research has often relied on younger "aged" models that fail to capture the complexity of aging in humans (Kim et al., 2021). Focusing on mice equivalent in age to elderly humans is particularly noteworthy and provides a more realistic assessment of therapeutic potential, offering valuable insights into the possibility of translating the approach to humans.

While the findings represent a significant advance, they also raise important questions. Can the benefits of intermittent fasting be replicated in humans through NAD^+^ supplements or by modulating gut bacteria to provide alternative treatments for those not able to follow strict dietary regimens? How do interactions between osteoprogenitors and other microenvironment cells, such as immune cells, influence the observed outcomes? Would similar effects occur in younger individuals or other tissues? Future studies must address these gaps to accelerate the therapeutic potential of these approaches.

The significance of these findings extends beyond bone repair by suggesting the potential for restoring functionality to other aged tissues through targeting metabolic and cellular pathways. This raises intriguing possibilities for applying these strategies to tissues like skin or muscle, which also lose regenerative capacity with age.

Looking ahead, the findings of Reeves et al. open pathways for therapies combining localised treatments, like Wnt3a ligands, with systemic interventions targeting the metabolism. If successfully applied to humans, these therapies could transform care for elderly patients, enhancing fracture healing and, as a result, quality of life.
